# Frequency and antimicrobial resistance pattern of non lactose fermenting gram negative rods in neurosurgical patients outlining aminoglycoside resistance genes

**DOI:** 10.1186/s12866-025-04422-5

**Published:** 2025-10-28

**Authors:** Sylvana Nady Gaber, Aya Ahmed Abdullah, Ashraf Abdel-Latif Osman, Mahmoud A.F. Khalil, El Shaimaa Gomaa Ali

**Affiliations:** 1 Department of Medical Microbiology and Immunology, Faculty of Medicine, Fayoum University, Fayoum, Egypt; 2 Department of Neurosurgery, Faculty of Medicine, Fayoum University, Fayoum, Egypt; 3 Department of Medical Microbiology and Immunology, Faculty of Pharmacy, Fayoum University, Fayoum, Egypt

**Keywords:** Non-Fermenting Gram-Negative rods, Hospital-acquired infections, Aminoglycosides resistance, Neurosurgical patients

## Abstract

**Background:**

Non-Fermenting Gram-Negative Rods (NFGNRs) are one of the major causes of hospital-acquired infections (HAIs) with high levels of antibiotic resistance. The patients following neurosurgery are continuously at risk of HAIs due to the severity of the brain insult. We aimed to assess the frequency and the characterization of antimicrobial resistance patterns among NFGNR-causing HAIs isolated from neurosurgical patients with special reference to the aminoglycoside resistance.

**Methods:**

A cross-sectional study was conducted on the hospitalized neurosurgical patients at Fayoum University Surgical Hospital, Egypt. The demographic characteristics and clinical data of patients were reported. The HAI definition was used by the Centers for Disease Control and Prevention (CDC). From 120 patients, one hundred and thirty-one samples were obtained and cultured on an appropriate media. NLFGNRs were identified using standard microbiological methods and VITEK2. Antimicrobial susceptibility testing was evaluated using the disc diffusion method. PCR was used to detect the aminoglycosides-resistant genes. Simple descriptive statistical analysis for results was done using statistical software, SPSS version 24. The chi-square and Fisher’s exact test for the bivariate relationship were performed.

**Results:**

One hundred and forty-six strains were isolated. NFGNRs represented [63/146 (43.1%)]. The most frequent NFGNRs were *Pseudomonas aeruginosa* ***(****P. aeruginosa)* (66.7%) and *Acinetobacter baumannii* (*A. baumannii) (*33.3%). 63.5% of NFGNR were resistant to aminoglycosides. *P. aeruginosa* exhibited a higher rate of resistance to gentamicin (62%), amikacin (59.5%), and meropenem (57.1%). In addition, *A. baumannii* was resistant to gentamicin (76.1%), amikacin (62%), and meropenem, ceftazidime (57.1%) for both. *armA* gene was the most detected aminoglycoside resistance gene (97.7%), followed by *aac(3ʹ)-IIa* (59.1%).

**Conclusions:**

We reported a high prevalence of NFGNRs (43.1%) causing HAIs among neurosurgical patients with increased antibiotic resistance for gentamicin. This makes a significant challenge for continued surveillance of antibiotic resistance and the improvement of the antimicrobial stewardship program to lower the frequency of NFGNR infections among neurosurgical patients.

## Introduction

Gram-negative rods non- fermenting are aerobic groups of bacteria that are unable to ferment sugars and derive energy from simple carbohydrates using an oxidative pathway [1]. They are a heterogeneous group of bacteria that includes *Pseudomonas* species (spp.), *Acinetobacter* spp., *Burkholderia* spp., and *Stenotrophomonas maltophilia (S. maltophilia).* They account for approximately 15% of all gram-negative (GN) bacterial infections [2]. The ability of NFGNRs to survive under a wide range of environmental conditions has allowed them to persist in the hospital environment, such as respirators, sinks, nebulizers, dialysate, and other devices [1]. NFGNRs could be recovered from the body surface of healthcare workers. They are frequently involved in nosocomial infections and are known to increase mortality [3]. Neurosurgical patients who have traumatic brain injuries, ischemic strokes, or spontaneous hematomas are susceptible to post-neurosurgical infections like systemic infections, meningitis, ventriculitis, and nosocomial infections [4]. Among neurosurgical patients, the rate of nosocomial infections reaches up to 36%. 40% of patients develop at least one infection, pneumonia being the commonest (38%), followed by urinary tract infections (UTIs) (9%) and surgical site infections (9%) [5]. It was reported that the NFGNRs infection rate is increasing after neurosurgery due to multiple drug resistance (MDR) activity of these pathogens and long ICU stays [4]. In NFGNRs, the antibiotic resistance mechanisms originate from antibiotic-inactivating enzymes expression or non-enzymatic paths (such as the expression of efflux pumps or target modifications) [6]. Central nervous system bacterial infections are serious, which may cause disability of the patients or death. Many classes of antibiotics are used for treatment, like beta-lactam antibiotics, vancomycin, linezolid, aminoglycosides, daptomycin, trimethoprim/sulfamethoxazole, doxycycline, and polymyxin B [7]. In recent years, due to the increasing frequency of intracranial device usage and MDR pathogens, treatment of cranial infections with intraventricular aminoglycosides has become progressively common. In addition, aminoglycosides suppress bacterial growth for a long time after administration, as they show concentration-dependent killing [8]. Aminoglycosides are highly powerful broad-spectrum antibiotics used to treat life-threatening infections. They primarily act by disrupting protein synthesis, because they bind to the aminoacyl site of the 16 S ribosomal RNA within the 30 S ribosomal subunit, leading to misinterpretation of the genetic code and mistranslation [9]. Because aminoglycoside antibiotics have commonly been used to target gram-negative bacteria (GNB), the emergence of MDR strains resulted in major transmission of these pathogens around the world (10). In a study in the USA done on 1349 clinical isolates of GNB, it was observed that 90% of resistance was to amikacin [9]. In Iran, Azimi et al. [11] revealed that phenotypic and genotypic aminoglycoside resistance among GNB was quite high. Also in Texas, Akers et al. [12] showed that the aminoglycoside resistance among NFGNR was 93% to gentamicin and 87% to tobramycin. In Egypt, a significant prevalence rate of aminoglycoside resistance among clinical isolates of GNB is described by different previous studies [13, 14]. The commonest aminoglycoside resistance mechanism is aminoglycoside enzymatic modification and 16 S rRNA methylation. Aminoglycoside modification enzymes (AMEs) consist of 3 classes: adenylation nucleotidyl transferases (ANT), acetylation acetyltransferases (AAC), and phosphorylation phosphotransferases (APH) [10]. 16 S rRNA methylation blocks the aminoglycoside binding site by methylating the nucleotide residues on the 16 S rRNA and thus leads to resistance [15]. The rising drug resistance among NFGNRs can lead to the urgent need for close monitoring of the antimicrobial susceptibility profile of these organisms [2]. In the current study, we aimed to assess the frequency and the characterization of resistance patterns among NFGNR-causing HAIs isolated from neurosurgical patients with special reference to the aminoglycoside resistance.

## Methods

 A cross-sectional study was conducted on the hospitalized neurosurgical patients in Fayoum University Surgical Hospital, Egypt, from April 2022 to December 2022. Through the study period, the neurosurgical patients who were aged 16 years or older with symptoms and signs of infections (such as fever >37.5 °C, leukocytosis, or pus from a wound) occurring more than 48 h following hospital admission were included in the study [16]. Exclusion criteria included patients who were suspected of having a cranial infection preceding the neurosurgical procedure or patients with symptoms or signs of infection within 48 h after admission.

One hundred and thirty-one samples were obtained from the patients who were admitted to the neurosurgical department and the Neurosurgical intensive care unit (NICU). The procedures of the present study were permitted by the Institutional Ethics Committee (Fayoum University Ethics Committee) NO: M590 (Session no.93, dated 4–2022). In compliance with Helsinki’s medical guidelines, this study has been conducted. The patient’s or their close relative’s written consents were obtained after they were briefed about the study’s objectives, and the demographic data of each patient was collected, which includes name, age, and gender.

### Patients

 One hundred and twenty inpatients and NICU residents who followed the inclusion criteria were included in the study. The clinical characteristics involved the site of infection, the presence of underlying diseases, and risk factors (diabetes, immunocompromising diseases, and frequent use of antibiotics or immunosuppressing drugs).

### Collection of samples

 Eighty urine samples, 34 endotracheal aspirates, 11 wound swabs, and 6 blood samples were collected. Samples were transported (within two hours) to the Medical Microbiology and Immunology Department, Faculty of Medicine, Fayoum University. All specimens were collected aseptically and given serial numbers to code each specimen with careful labeling. Samples were processed according to the standard microbiological methods [5].

### Isolation and identification of microorganisms

 Specimens were inoculated onto different culture media, including Nutrient agar, MacConkey agar, Blood agar, and CLED (Oxoid, The United Kingdom), under aerobic conditions and incubated at 37 °C for 24 h. The blood culture incubation system [BactT-Alert 3D (BioMérieux, Inc., Durham, NC, USA**)]** was used for the isolation of bacteria from the blood cultures then sub-cultured onto the blood agar and MacConkey agar and incubated at 37 °C for 24 h. The isolated organisms were identified by using the VITEK2 Compact 15 (BioMérieux, Inc., Durham, NC, USA**).**

### The antimicrobial susceptibility testing

 The antimicrobial susceptibility test was performed by the Kirby-Bauer disc diffusion method. The used antibiotic discs were aminoglycoside; amikacin (30 µg), gentamicin (30 mg), third-generation cephalosporin; ceftazidime (30 µg), ceftriaxone (30 µg), cefotaxime, (30 µg), fourth-generation cephalosporin; cefepime (30 µg), carbapenem; meropenem (10 µg) β-lactamase inhibitor; piperacillin-tazobactam, (100/10 µg), ampicillin/sulbactam (10/20 µg), and quinolone; ciprofloxacin (5 mg) (Oxoid Ltd., UK). The agar was incubated at 37 ˚C for 24 h. The zones of growth inhibition around each of the antibiotic discs were measured to the nearest millimeter. Sensitivity zone measurements were according to CLSI guidelines [17]. For colistin, the microdilution method was used, with the determination of the minimum inhibitory concentration (MIC) [17]. As a control strain, *P. aeruginosa* ATCC 27,853 and *Escherichia coli (E. coli)* ATCC 25,922 were used. For colistin, we employed *E. coli* NCTC 13,846.

The isolates were preserved in glycerol broth at − 80 ° C for further analysis. MDR organisms are defined as acquired non-susceptibility to at least one agent in three or more antimicrobial categories. Extensively drug-resistant (XDR) organisms are well-defined as non-susceptibility to no less than one agent in all but two or fewer antimicrobial categories (i.e., bacterial isolates persist susceptible to only one or two categories), and Pan-Drug Resistant (PDR) bacteria were defined as non-susceptibility to all agents in all antimicrobial categories [18]. Any isolates resistant to at least one of the aminoglycoside agents were considered aminoglycoside-resistant isolates. The aminoglycoside-resistant isolates were further genetically examined by identification of aminoglycoside resistance genes among NFGNRs.

### DNA extraction and genetic characterization of aminoglycoside resistance genes

Genetic characterization of aminoglycoside resistance genes *(aac (3)-ll*,* rmtB*,* and armA*,* aacC1*,* aadB*,* aadA1*,* and aphA6)* was performed by conventional monoplex PCR. Primers used for gene detection are shown in Table 1 [14, 19].

**Table 1:** Primer’s sequence of the studied genes

Gene Name	Sequence (5ʹ–3ʹ)	Amplicon Size (bp)	Reference
*aac(3ʹ)-IIa*	F: ATATCGCGATGCATACGCGG R: GACGGCCTCTAACCGGAAGG	877	[19]
*armA*	F: CCGAAATGACAGTTCCTATC R: GAAAATGAGTGCCTTGGAGG	846	[19]
*rmtB*	F: ATGAACATCAACGATGCCCTC R: CCTTCTGATTGGCTTATCCA	769	[19]
*aacC1*	F : ATG GGC ATC ATT CGC ACA TGT AGG R: TTA GGT GGC GGT ACT TGG GTC	456	[14]
*aadB*	F: ATG GAC ACA ACG CAG GTC GC R: TTA GGC CGC ATA TCG CGA CC	534	[14]
*aadA1*	F: ATG AGG GAA GCG GTG ATC G R: TTA TTT GCC GAC TAC CTT GGT G	792	[14]
*aphA6*	F: ATG GAA TTG CCC AAT ATT ATT C R: TCA ATT CAA TTC ATC AAG TTT TA	797	[14]

*bp* Base pair, *aac(3ʹ)-II*a aminoglycoside acetyltransferase, *armA* aminoglycoside ribosomal metylase, *rmtB* ribosomal methylase*, aacC1* aminoglycoside acetyltransferase, *aadB* aminoglycoside nucleotidyltransferase 2, *aadA1* aminoglycoside nucleotidyltransferase 3, *aphA6* aminoglycoside phosphotransferase

#### DNA extraction

Was performed by the heating method as follows: one to two colonies of isolated resistant organisms were added to 750 µl of distilled water. Heating at 95˚C for 5 min. Centrifugation occurred at 12,000 rpm for 10 min, then the supernatant was used. This method has the advantage of being quick, cheap and direct [20].

The purified DNA samples were assessed for DNA yield using a NanoDrop (ND-1000) spectrophotometer (NanoDrop Technologies, Inc., Wilmington, USA), then stored at −80̊ C.

#### Monoplex PCR detection of aac-(3’)-lla, rmtB, and armA genes

Was performed using the Long Gene A200 Gradient Conventional PCR Thermal Cycler system under the following conditions: Initial denaturation at 95˚C for 5 min; 31 PCR cycles of denaturation at 95˚C for 40 s, annealing 55˚C for50˚C, extension at 72˚C for 60 s, and elongation at 72˚C for10 min.

#### Monoplex PCR detection of aphA6, aacC1, aadB, and aadA1 genes

Was performed under the following conditions: Initial denaturation at 95˚C for 5 min; 31 PCR cycles denaturation at 95˚C for 50 s, annealing 52˚C for 50˚C, extension at 72˚C for 60 s, and elongation at 72˚C for7 min. The electrophoresis technique was used to detect PCR products on 1% agarose gel in 1x buffer 50 ml (TE Buffer, Thermo Fisher Scientific Inc.US) and examined beneath UV light (Cleaver Scientific, UK).

### Sample size

The sample size was determined by the total number of HAI cases identified during the study period. One hundred and twenty hospitalized patients (from a total of 488 hospitalized patients) with hospital-acquired infections from April 2022 to December 2022 were included in this cross-sectional study.

### Statistical analysis

 The statistical package for social science (SPSS version 24) was used for data analysis. Simple descriptive statistics (arithmetic mean and standard deviation) were used for summary and normal quantitative data. The bivariate relationship was displayed in cross-tabulation, and a comparison of proportions was performed using the chi-square and Fisher exact test where appropriate. The level of significance was set at a probability (P) value ≤ 0.05.

## Results

### Demographic and clinical characteristics of the studied patients

 Among 120 neurosurgical patients, males [95/120 (79.2%)] were more than females [25/120 (20.8%)], with a mean age of 47.5 years $\:\pm\:$ 18 yrs Table 2. Brain hemorrhage [80/120 (66.6%)] was the most common presentation among the studied patients and the greatest co-morbidity was hypertension, which represented (20.8%). Table 3.

**Table 2:** The demographic characteristics of the studied patients

		Mean	SD
Age		47.5	18
		N	%
Sex	Female	25	20.8%
	Male	95	79.2%
Total		120	100%

N Number of patients, % Percentage

**Table 3:** Causes of admission and co-morbidities among the studied patients (No. 120 patients)

Cause of admission	N	%
Brain hemorrhage	**80**	**66.6%**
Brain tumor	18	15%
Stroke	12	10%
Co-morbidity		
HTN	25	20.8%
DM	21	17.5%
Cardiac diseases	7	5.8%
HBV	3	2.5%
Chronic kidney disease	1	0.8%
HCV	1	0.8%

*N* Number of patients, *HTN* Hypertension, *DM* Diabetes mellitus, *HBV* Hepatitis B virus, *HCV* Hepatitis C virus, *% P*ercentage

### Clinical specimens from the studied patients

 out of 131 specimens, [107/131 (81.6%)] specimens showed bacterial growth with 146 isolates. [37/107 (34.6%)] specimens were polymicrobial, while [70/107 (65.4%)] specimens were monomicrobial. The most common collected specimens were urine [80/131(61.1%)], followed by endotracheal aspirate [34/131 (26%)] then wound [11/131 (8.4%)], and blood [6/131 (4.6%)].

### Distribution of microorganisms among different specimens

Among 146 isolates, *P. aeruginosa* had the highest prevalence [42/146 (28.7%)], followed by *Klebsiella* spp. [33/146 (22.6%)] and *A. baumannii* [21/146(14.4%)]. Our samples also showed growth of *E. coli* [16/146 (11%)], and *Staphylococcus* spp. [15/146(10.3%)]. *P. aeruginosa* and *A. baumannii* were most frequently isolated from urine samples, followed by endotracheal aspirate Table 4.

**Table 4:** Distribution of microorganisms isolates in different specimens

Microorganism	Specimen				
	Urine N (%)	Endotracheal aspirate N (%)	Wound N (%)	Blood N (%)	Total N (%)
*P. aeruginosa*	23(28.8%)	16(32%)	0(0.0%)	3(60.0%)	42 (28.7%)
*A.baumannii*	11(13.8%)	9(111.3%)	1(9.1%)	0 (0.0%)	21(14.4%)
*Klebsiella *spp.	13(16.3%)	19(38.0%)	0(0.0%)	1(20.0%)	33(22.6%)
*E.coli*	12(15.0%)	3(6.0%)	1(9.1%)	0(0.0%)	16(11.0%)
*Staphylococcus *spp.	3(3.75%)	2(4.0%)	9(81.8%)	1(20.0%)	15(10.3%)
*Candida* spp*.*	18(22.5%)	1(2%.0)	0(0.0%)	0(0.0%)	19(13.0%)
Total	80(100%)	50(100%)	11(0.0%)	5(100%)	146 (100%)

*Spp S*pecies, *P*.*aeruginosa *Pseudomonas aeruginosa, *A. baumannii*Acinetobacter baumannii,* E. coli *Escherichia coli

Our finding revealed that the prevalence of NFGNRs was: [63/146 (43.1%)]. *P. aeruginosa* was [42/63 (66.7%)] while *A. baumannii* was [21/63 (33.3%)]. NFGNR isolates were higher in males [47/63 (74.6%)] as compared to females [16/63 (25.4%)].

Among the studied patients, NFGNRs represented [63/120 (52.5%)]: *P. aeruginosa* isolates were [42/120 (35%)], while *A. baumannii* isolates were [21/120 (17.5%)].

### Antimicrobial susceptibility testing of non-fermenting gram-negative rods

 Regarding *P. aeruginosa*, isolates exhibited a higher rate of resistance to gentamicin (62%) than amikacin (59.5%), and meropenem (57.1%). In addition, *A. baumannii* was resistant to gentamicin (76.1%), amikacin (62%), and meropenem, ceftazidime, and ciprofloxacin (57.1%) for all Table 5. Also, 63.5% of NFGNRs were resistant to aminoglycosides. (46%) of isolated NFGNR were XDR. Of the *P. aeruginosa* isolated, 45.2% were XDR, and 47.6% of the total *A. baumannii* isolated were XDR.

**Table 5:** Antimicrobial susceptibility testing of none-fermenting gram negative bacilli

Antibiotic	*P.aeurginosa* (n=42)			*A.baumannii *(n=21)		
	R (%)	I	S	R (%)	I	S
Aminoglycosides						
Gentamicin	26 (62%)	**_**	16	16(76.1%)	1	4
Amikacin	25(59.5%)	1	16	13(62%)	_	8
B lactams						
Ceftazidime	22(52.3%)	10	10	12(57.1%)	6	3
Meropenem	24(57.1%)	9	9	12(57.1%)	6	3
Cefepime	21(50%)	10	11	11(52.3%)	8	2
Pipracillin/tazobactam	16(38%)	8	18	8(38%)	_	13
Ampicillin/sulbactam	NT			10(47.6%)	8	3
Ceftriaxone	NT			10(47.6%)	9	2
Cefotaxime	NT			11(52.3%)	9	1
Others						
Ciprofloxacin	23(54.7%)	10	9	12(57.1%)	6	3
Colistin	-	-	42	-	-	21

*N* Number of isolates, *R* Resistant, *I* Intermediate, *S* Sensitive, *CLSI* The Clinical and Laboratory Standards Institute, *NT* Not tested by CLSI

### Frequency of aminoglycoside resistance genes among non-fermenting gram-negative rods

 Our findings showed that: the *armA* gene was the most detected gene among aminoglycosides-resistant isolates [43/44 isolates (97.7%)], followed by *aac (3ʹ)-IIa* [26/44 isolates (59.1%)], and *aacC1 *[24/44 (54.5%)] isolates Table 6.

**Table 6:** =Frequency of aminoglycoside resistance genes among none-fermenting gram-negative bacilli (N: 44 isolates)

Gene	Positive
	N (%)
*armA*	**43 (97.7 %)**
*aac(3ʹ)-IIa*	**26 (59.1 %)**
*aacC1*	24 (54.5 %)
*rmtB*	22 (50 %)
*aphA6*	18 (40.9 %)
*aadA1*	16 (36.4 %)
*aadB*	1 (2.3 %)

*N* Number of isolates, *%* Percentage, *aac(3ʹ)-IIa* aminoglycoside acetyltransferase, *armA* aminoglycoside ribosomal methylase,*rmtB* ribosomal methylase, *aacC1* aminoglycoside acetyltransferase, *aadB* aminoglycoside nucleotidyltransferase 2*, aadA1* aminoglycoside nucleotidyltransferase 3, *aphA6* aminoglycoside phosphotransferase

The present study found that the prevalence of the positive *aacC1* gene was higher in amikacin-resistant isolates as compared to non-resistant isolates (60.5% vs. 16.7%) *(P-value* = 0.045). Also, the prevalence of the positive *armA* gene was higher in gentamicin**-**resistant isolates as compared to non-resistant isolates (100% vs. 50%) (*P-value* ˂ 0.001). And the prevalence of the positive *rmtB* gene was higher in both gentamicin and amikacin-resistant isolates as compared to non-resistant isolates (58.3% vs. 12.5%) (*P-value* = 0.046) Table 7. *P. aeruginosa* isolates (66.7%) had a statistically significant higher proportion of positive *rmtB* gene than *A. baumannii* isolates (23.5%) (*P-value* = 0.005) Table 8.

**Table 7:** Relation between aminoglycoside resistance genes and aminoglycoside resistance

		AK				GN				Both CN & AK			
		Resistant		Not resistant		Resistant		Not resistant		Resistant		Not resistant	
		N	%	N	%	N	%	N	%	N	%	N	%
*P*-value		0.598				<0.001*				0.158			
aacC1	Positive	23	60.5%	1	16.7%	24	57.1%	0	0.0%	23	63.9%	1	12.5%
*P*-value		0.045*				0.201				0.015*			
aphA6	Positive	14	36.8%	4	66.7%	17	40.5%	1	50.0%	13	36.1%	5	62.5%
*P*-value		0.208				1.000				0.240			
aadA1	Positive	13	34.2%	3	50.0%	15	35.7%	1	50.0%	12	33.3%	4	50.0%
*P*-value		0.652				1.000				0.434			
armA	Positive	37	97.4%	6	100.0%	42	100.0%	1	50.0%	36	100.0%	7	87.5%
*P*-value		1.000				<0.001*				0.032*			
rmtB	Positive	21	55.3%	1	16.7%	22	52.4%	0	0.0%	21	58.3%	1	12.5%
*P*-value		0.185				0.488				0.046*			
*aac(3ʹ)-IIa*	Positive	23	60.5%	3	50.0%	24	57.1%	2	100.0%	21	58.3%	5	62.5%
*P*-value		0.676				0.505				1.000			

*P*-value < 0.05 is considered significant

*N* Number of organisms*Significant, AK amikacin, CN gentamicin, *aac(3ʹ)-IIa* aminoglycoside acetyltransferase,* armA* aminoglycoside ribosomal methylase, *rmtB* ribosomal methylase, *aacC1* aminoglycoside acetyltransferase, *aadB* aminoglycoside nucleotidyltransferase 2, *aadA1* aminoglycoside nucleotidyltransferase 3*, aphA6* aminoglycoside phosphotransferase

**Table 8:** Relation between aminoglycoside resistance genes and isolated organism

		BACTERIA				*P-value*
		*A.baumannii*		*P. aeruginosa*		
		N	%	N	%	
*aacC1*	Positive	9	52.9%	15	55.6%	0.865
*aphA6*	Positive	8	47.1%	10	37.0%	0.510
*aadA1*	Positive	5	29.4%	11	40.7%	0.477
*arm A*	Positive	16	94.1%	27	100.0%	0.202
*rmtB*	Positive	4	23.5%	18	66.7%	0.005*
*aac(3ʹ)-IIa*	Positive	7	41.2%	19	70.4%	0.055
*aadB*	Positive	0	0.0%	1	3.7%	1.000

*P*-value < 0.05 is considered significant,

*N* Number of organisms*Significant, *aac(3ʹ)-IIa* aminoglycoside acetyltransferase, *armA* aminoglycoside ribosomal methylase, *rmtB* ribosomal methylase, *aacC1* aminoglycoside acetyltransferase, *aadB* aminoglycoside nucleotidyltransferase 2, *aadA1* aminoglycoside nucleotidyltransferase 3, *aphA6* aminoglycoside phosphotransferase

## Discussion

 Non-fermenting gram-negative rods cause various nosocomial infections, including pneumonia, septicemia, wound infections, meningitis, and UTIs. The extensive use of antibiotics, especially aminoglycosides leads to increased numbers of antimicrobial-resistant bacteria. This development makes the treatment of patients infected with these pathogens difficult and consequently increases morbidity and mortality [21]. Neurosurgical intensive care units always play an increasingly important role in neurosurgical patients, where postoperative ICU monitoring is widely considered essential following cranial procedures [22]. In the present study we aimed to assess the frequency of NFGNR-causing HAIs among neurosurgical patients, and our findings reported that the prevalence of NFGNRs was 43.1% with *P. aeruginosa* (66.7%) being the most predominant isolate, followed by *A. baumannii* (33.3%). The current results agree with Lakhani et al. [23], who revealed the most common isolate was *Pseudomonas* spp. (42%), followed by *A. baumannii* (31%), and are higher than the Indian report carried out by Reddi and Israel [24]., who found that *P. aeruginosa* (49%), was followed by *A. baumannii* (19%) among (49%) isolated NFGNB. The results differed from Chaudhury et al. [25], who stated that the highest number of isolates was *A.baumannii*, comprising (27.69%) followed by *S. maltophilia* (21.53%), and *P. aeruginosa* (13.84%). Also, Ozenen et al. [26], reported that *Acinetobacter* spp. (35.9%) was the most common isolate, followed by *Pseudomonas* spp. (34.4%). These diversities could be explained by the difference in the site of samples and the difference in predominant clones.

Non-fermenting gram-negative rods unfortunately prompt many resistance mechanisms to antimicrobials. There has been augmented resistance to the already small number of antimicrobials that are frequently used to treat NFGNRs. One of the reasons thought to be accounting for this rise is the increased use of broad-spectrum antibiotics [25]. Concerning antibiotic resistance in NFGNRs, *A. baumannii* showed the highest resistance to gentamicin (76.1%), and 47.6% were XDR. *P. aeruginosa* showed the highest resistance to gentamicin (62%), and 45.2% were XDR. El-Far et al. [20] showed similar results, as *P. aeruginosa* isolates had the highest resistance (97%) to aminoglycosides and (57.6%) were XDR. Chawla et al. [27] differ from these findings, as the highest drug resistance rates of *P. aeruginosa* and *A. baumannii* were (89.6%) for ampicillin, and among *P. aeruginosa* isolates, (9.8%) were XDR. Of 28 isolates of *A. baumannii* (35.7%) were XDR. On the other hand, Porbaran and Habibipour [28] disagreed, as *P*. *aeruginosa* isolates had the highest drug resistance to ciprofloxacin (72%) and gentamicin (66%) and had 14% XDR strains, while *A. baumannii* isolates had the highest drug resistance to ciprofloxacin (87.14%) and gentamicin (77.14%) and had (15.71%) XDR strains. The distinct resistant pattern among the different studies could be influenced by variations in environmental factors of different settings, unrestricted use of antibiotics, and lack of awareness among clinicians about using broad-spectrum antibiotics.

A comprehensive understanding of aminoglycoside resistance genes, prevalence, and spreading is needed. Consequently, to better recognize the resistance status of aminoglycoside and the frequency of the resistance genes we were concerned with, we used genotypic detection of aminoglycoside resistance genes. Our findings showed that *armA* (94.1%) was the most common gene among *A. baumannii*, followed by *aphA6* (47.1%). For *P. aeruginosa*,* armA* (100%) had the highest prevalence, followed by *aac(3’)-II* (70.4%) and *rmtB* (66.7%). EL Sheredy et al. [29] found that *A. baumanni* had *aphA6* (86%) as the highest aminoglycoside resistance gene, followed by *armA* (83%). Also, El-Far et al. [20] partially agreed with our results, as they showed a predominance of *rmtB* (51.5%), followed by *armA* (9%) among *P. aeruginosa.* Kishk et al. [14] determined that among *A. baumannii* isolates the predominant AME gene was the aacC*1* gene detected in (40%), *aphA6* in (31.4%), and *addA1* in (14.2%). But Panahi et al. [30] results, were different, as the most prevalent aminoglycoside resistance genes were *ant (2″)-Ia* and *aac (6′)-Ib*, observed in 85% and 71.2%, respectively, among *P. aeruginosa*. Khurshid et al. [31] found that *aphA6* (74.1%) had the highest prevalence of aminoglycoside resistance genes among *A. baumannii*, followed by *aadB* (59.4%) and *armA* (28%).

Zhang et al. [10] revealed that the most common AME genes differ between countries in the United States (USA). *P. aeruginosa* had *aac (6′)-Ib*,* aac (3)-IV*,* ant (2″)-Ia*,* and aph (3′)-Ia.* China paid more attention to the detection of 16 S rRNA methylase genes with AME genes. El-Far et al. [20] stated that the 16 S rRNA methylase gene was the main aminoglycoside-resistant gene in *P. aeruginosa* isolates, with *rmtB* being the chief gene.

Our results found that: *P. aeruginosa* gentamicin resistant isolates had a statistical significant high proportion of positive *aac (3’)-II*a gene (69.2%). Zhang et al. [10] revealed that there were significant correlations between *aph(3′)-VIa* and amikacin resistance reported in *A. baumannii and P. aeruginosa* isolates, and *ant (2)-Ia* seemed to be an important determinant of resistance to gentamicin and tobramycin. Considering these differences, it seemed that the aminoglycoside resistance genes were disseminated among numerous groups of strains. Thus, these data could reflect a widespread occurrence and variations in HAIs NFGNR isolates in several regions. Also, it might be due to variations in the usage of aminoglycosides in different geographical areas.

Regarding demographic and clinical data of our patients, Agarwal and his colleagues [32], in agreement with our results, found that among neurosurgical patients: (60.9%) were males and (39.1%) were females, and the mean age was 30.3 years. Furthermore, our results revealed that (66.6%) of patients presented with brain hemorrhage, and the most common co-morbidity was hypertension (20.8%). Ehlers and co-workers [22] had (66.3%) patients with hypertension. While Agarwal and his colleagues [32] reported that the most common underlying disease was intracranial tumors (47.6%), followed by aneurysms (19%). That is probably due to different geographical distribution and different healthcare services.

In India, Menon et al. [33] found that urine samples (78.4%) were the most collected samples, and this is matched with our findings in which urine specimens (61.1%) were the most frequently collected specimens. In addition, Agarwal and his colleagues [32] revealed that UTI was most common (71.4%), followed by blood-stream infection (28.5%). On the other hand, Salmanov et al. [16] found that wound infections (53.2%) and respiratory infections (17.3%) were the most occurring HAIs. Also, Busl [34] reported that pneumonia was the most common (38%), followed by UTI (9%). This difference in HAIs distribution may be due to the variation in treatment protocols, infection prevention, and control measures.

*P. aeruginosa* had the uppermost prevalence (28.7%) than *Klebsiella* spp. (22.6%) and *A. baumannii* (14.4%). That differs from La Fauci et al. [35], who found that *A.baumannii* (42.6%), *Klebsiella* spp. (23.3%), and *P. aeruginosa* (14. 2%) were the most common isolates. Also, Russo et al. [36] reported that *S.aureus* (14.4%), *Candida albicans* (9.5%), and *E. coli* (9.2%) were the most common organisms.

### Limitations of the study

Our study is a single-center study, as our country, EL Fayoum, is a small governorate and has only one center of neurosurgical operations and a neurosurgical intensive care unit of a university hospital. In the future, we plan to do a multicentric study from different countries. The sample size is low, which decreases the statistical power of the analyses, so we recommended a large study on a large number of patients.

## Conclusions

The prevalence of NFGNR infections was high (43.1%) among our neurosurgical patients. *P. aeruginosa* had the highest prevalence, followed by *A. baumannii*. The high prevalence of 16 S RMTase confers resistance to all aminoglycosides. Therefore, integrated activities in the form of regular surveillance of antimicrobial resistances with an improvement of antibiotic stewardship are needed to control aminoglycoside resistance before it becomes a threatening occasion.

## Data Availability

All data generated or analyzed during this study are included in this article.

## Abbreviations

NFGNR: Non-Fermenting Gram Negative Rods
HAIs: Hospital-acquired infections
XDR: Extensively drug-resistant
GN: Gram Negative
ICU: Intensive care unit
MDR: Multidrug-resistant
AMEs: Aminoglycoside modifying enzymes
AAC: Acetylation acetyltransferases
ANT: Adenylation nucleotidyl-transferases
APH: Phosphorylation phosphotransferases
16s-RMTase: 16 S Ribosomal RNA methyltransferases
NICU: Neurosurgical Intensive care unit
MIC: Minimum inhibitory concentration
PDR: Pan-Drug Resistant
ND: NanoDrop
E. coli: Escherichia coli
P. aeruginosa: Pseudomonas aeruginosa
A. baumannii: Acinetobacter baumannii
bp: Base pair
aac(3ʹ)-IIa: Aminoglycoside acetyltransferase
armA: Aminoglycoside ribosomal methylase
rmtB: ribosomal methylase
aadB: Aminoglycoside nucleotidyltransferase
aadA1: Aminoglycoside nucleotidyltransferase
aphA6: Aminoglycoside phosphotransferase
AK: Amikacin
CN: Gentamicin
N: Number of isolates
%: Percentage
CLSI: The Clinical and Laboratory Standards Institute
UTIs: Urinary tract infections
SPSS: Statistical package for social science
S. aureus: Staphylococcus aureus
S. maltophilia: Stenotrophomonas maltophilia
USA: United States
